# Effects of visual thermal cues on thermal perception and thermoregulation while cycling in virtual reality

**DOI:** 10.1038/s41598-026-62664-3

**Published:** 2026-08-10

**Authors:** Martin Kocur, Adriana Chaparro-Gonzalez, Jonaris Delgado, Joshua Malam, Cheyenne Ivey, Kale Lawhorne, Nyah McFarlane, Sergi Garcia-Retortillo, Valentin Schwind, Niels Henze

**Affiliations:** 1 Department of Psychology, University of Central Florida, Orlando, 32816 USA; 2 College of Nursing, University of Central Florida, Orlando, 32827 USA; 3 Media Informatics, Stuttgart Media University, 70569 Stuttgart, Germany; 4 Department of Computer Science, University of Tuebingen, 72074 Tuebingen, Germany

**Keywords:** Neuroscience, Physiology, Psychology, Psychology

## Abstract

Thermal perception and regulation are essential for physical exercise. The hue-heat hypothesis suggests that an environment’s color temperature, e.g., warm reddish lighting or cold bluish lighting, affects thermal perception and regulation. Virtual reality (VR) enables immersion in any environment. Previous work showed that a VR environment can affect how warm or cold users feel during sedentary VR experiences. However, it is unclear whether such visual thermal cues also influence physiological and perceptual responses during physical exercise. Therefore, we conducted a controlled VR experiment (n = 34) examining whether a virtual heater, either activated or deactivated, influences skin temperature, thermal perception, and perceived exertion while cycling for 20 minutes in each condition. Activation of the virtual heater resulted in significantly higher skin temperature over time and increased thermal sensation compared to the deactivated heater, despite no differences in perceived exertion and heart rate responses. Thereby, we show that visual thermal cues can indeed influence users’ thermal perception and thermophysiological responses during physical exercise. Our findings highlight the role of visual environmental cues in shaping thermoregulation during immersive physical exercises.

## Introduction

Thermal perception and regulation are crucial for physical exercise. It is widely known that performance in exercise is affected by the ambient temperature^1,2^. A large body of research shows that performance is impaired while cycling in the heat^1,3–5^. Racinais et al.^6^, for example, showed that cyclists had a reduced power output when cycling during an environmental temperature of 36.0$^\circ$C compared to 8.2$^\circ$C. While the exact underlying mechanisms still need to be explored^1^, previous work suggests that during exercise in the heat, an increase in body temperature directly influences central nervous functions^7^. Consequently, the central nervous system activates anticipatory protective mechanisms to naturally protect the body from overheating by reducing muscle activation^7^. In addition, higher body temperature increases skin blood flow to dissipate heat. This creates competition for blood between working muscles that need oxygen and the skin that needs cooling^8^. Hence, increasing the body temperature through ambient temperature adds additional thermal strain to the body beyond the heat produced during exercise, leading to higher perceived exertion and reduced performance^2,4^.

Previous work found that the body temperature can even be increased by visual cues without actually changing the ambient temperature. The hue-heat hypothesis posits that color temperature influences thermal perception, e.g., warmer colors result in warmer thermal perception^9^. Whether the hue-heat hypothesis holds has been debated for decades^10^, but Wang et al.^11^ recently concluded, based on a systematic review, that the results confirmed the long-held hypothesis. Accordingly, Takakura et al.^12,13^ showed that watching videos showing a desert on a regular display increased participants’ skin temperature while videos showing a snowy environment decreased their skin temperature. While the hue-heat hypothesis poses one explanation for such effects, the authors argue that classical conditioning causes them. Seeing videos of a hot desert activates associations of how it feels in that place. This cognitive process activates the thermoregulatory mechanisms one would experience in a hot environment, resulting in increased body temperature^13^. Thus, real and virtual environment’s color temperature indeed influence thermal perception and thermoregulation.

Virtual reality (VR) enables immersion in any possible environment. Consequently, previous work investigated whether virtual environments can also influence thermal perception. Multiple studies confirmed the hue-heat hypothesis for VR and found effects of color temperature on thermal perception^14–17^ and even skin temperature^18,19^. Wührl et al.^19^, for example, consistently found that being immersed in a fire virtual environment in VR increases skin temperature compared to being in an ice environment. These findings suggest that the visual characteristics of virtual environments must be carefully considered when designing VR experiences. However, previous studies examined static or sedentary scenarios, for example participants sitting for several minutes while being immersed in a fire or ice-themed virtual environment^18^. It, therefore, remains unclear whether these effects also occur and persist during sustained physical activity.

During physical activity, the body’s interoceptive signals, i.e., increased body temperature or elevated heart, cause the body to actively regulate body temperature through thermoregulatory mechanisms, e.g., blood flow regulation or sweating^20^. Those interoceptive signals during physical activity could interfere with the effects caused by visual thermal cues. As a result, it remains unclear whether the hue–heat hypothesis persists under such conditions. Leveraging physical activity therefore provides a promising approach to evaluate the robustness of the hue–heat hypothesis and to better understand how visual cues influence users’ temperature perception and regulation when increased physiological signals are present. From an applied perspective, understanding whether visual thermal cues influence thermal perception and regulation during physical activity is also important for VR designers and researchers. VR exercise applications frequently immerse users in environments with different thermal cues. These cues are not merely aesthetic but they can shape how users perceive heat and effort^18,19^. If visual thermal cues amplify perceived warmth or exertion during physical activity, they may inadvertently increase perceived heat, elevate body temperature, and intensify feelings of effort. This, in turn, could negatively impact user experience, comfort, and performance. Therefore, a deeper understanding of how color-based thermal cues interact with physiological responses during exercise can inform the design of VR environments that better support user well-being and optimize performance.

In this paper, we therefore conducted a controlled VR experiment in a repeated-measures design with 34 participants to examine the effects of visual thermal cues during physical exercise. Participants cycled in VR for 20 minutes at a constant workload (in watts) per condition, while a virtual heater was either activated or deactivated. We assessed skin temperature, thermal perception, perceived exertion, and heart rate. Results indicate that hand skin temperature increased significantly over time when the virtual heater was activated compared to the deactivated condition. Participants also reported higher thermal sensation. Differently from what was expected, no observable differences in perceived exertion or heart rate was found. These findings suggest that visual thermal cues can influence both thermoregulatory responses and subjective thermal perception even during exercise. Overall, our results highlight the importance of environmental design in exercise contexts, particularly for the development of VR-based fitness applications.

## Method

We aim to investigate the effects of visual thermal cues on physiological and perceptual responses during physical activity. Grounded on the hue-heat hypothesis^10,13,18,21^, we hypothesized that cycling during an activated virtual heater increases participants’ skin temperature and perceived warmth (H1). Due to the sensation of increased warmth and the resulting thermophysiological response, i.e., increased skin temperature, we also hypothesized that the perception of effort as well as the heart rate increases (H2).

### Participants

Previous work indicates that visual thermal cues in VR have medium to large effects on both thermal perception and skin temperature^18^. Assuming a medium effect size ($\eta _p^2 = 06$), a power analysis conducted in G*Power^22^ indicated that a sample size of N = 34 is required to achieve a power of *1-β* = .80 at an *α =.05* level. Therefore, we recruited 41 students from our university who participated in the study in exchange for course credit. To ensure that participation was voluntary, participants could earn course credit through alternative activities that did not involve participation in the study, including completing alternative assignments or taking part in other experiments. Three of the participants were used in our pilot study to fine-tune parameters such as the exercise intensity and the experimental procedure. Data from four participants were excluded from the final analyses due to technical issues (e.g., electrode detachment or signal loss). Hence, the final sample (n = 34) included 19 male participants (age: *M=21.26*, *SD=8.48*; height: *M=178.21* cm, *SD=8.94* cm; body weight: 74.90 kg, *SD=14.53* kg) and 15 female participants (age: *M=19.00*, *SD=1.36*; height: *M=166.85* cm, *SD=7.86* cm; body weight: 61.74 kg, *SD=8.58* kg). To assess the participants’ level of fitness, we administered the SPF questionnaire by Delignières et al.^23^ (Likert items ranging from 1 to 13) as part of the demographic questionnaire (*M=8.17, SD=1.70*). In addition, we calculated participants’ maximum HR (*M = 159.64, SD = 9.66*) using the age-predicted $HR_{max}$ equation (i.e., $208 - 0.7 \times \text {age}$)^24^. Participants were informed that they could withdraw or discontinue participation at any time without penalty. The experimental protocol was reviewed and approved by the University of Central Florida Institutional Review Board (IRB). All methods were carried out in accordance with IRB guidelines and regulations. All participants provided informed consent.

### Study design

We conducted the controlled experiment using a within-participants design to determine the effects of the independent variable Virtual Heater (*off* vs *on*) on the dependent variables skin temperature, thermal perception, and perceived exertion. In line with previous work^18,25,26^, we also measured heart rate, room temperature, and the sense of presence to control for possible confounders. We counterbalanced the order of the two conditions across participants so that half began with the first and the other half with the second condition. To normalize exercise intensity across participants, we followed the Steep Bicycle Protocol proposed by Northridge et al.^27^ and adjusted the cycling watt output using their body weight (e.g., 15 W for 50 kg participants and 30 W for 100 kg participants; see Table 1 for the distribution of Watts per body weight for all participants). In line with previous work^28^, participants cycled for 20 minutes per condition at a predefined target wattage. To ensure that the exercise intensity was identical for each heater condition, we used a generally low intensity (ranging from 15 [W] to 30 [W] depending on body weight) to remain within the aerobic threshold of 70% of the maximum HR and avoid the lactate turn point^29^. The ergometer was set to watt mode, allowing it to dynamically adjust resistance to maintain the specified power output, thereby ensuring a cadence-independent workload. We conducted a pilot study (n = 3) to fine-tune parameters such as the exercise intensity and monitor HR to determine whether the experiment causes the HR to remain below 70% of its maximum.

### Dependent variables

We took objective and subjective measures to determine the effects of the independent variable. Peripheral temperature changes capture vasoconstriction and vasodilation processes, which reflect the body’s cooling and warming strategies^30,31^. In line with Kocur et al.^18^, we therefore, continuously measured participants’ skin temperature on their right and left hand. Instead of assessing core body temperature, we followed prior work^18,31,32^ by measuring hand temperature as a more sensitive indicator of thermoregulatory responses. We additionally assessed participants’ thermal perception every 90 seconds using the Thermal Sensation Scale^33^. Participants rated their current thermal sensation in response to the question “How do you feel right now?” on a 7-point Likert scale ranging from cold to hot. We also measured thermal comfort using the Bedford Thermal Scale^34^ once at the end of each condition (“How would you rate your thermal comfort?”, using a 7-point Likert item ranging from too cold to too hot). We measured the perception of effort using the well-established Borg Ratings of Perceived Exertion (RPE) Scale^35^ ranging from 1 (no exertion at all) to 10 (maximum exertion). To measure the sense of presence in the virtual environments, we used the iGroup Presence Questionnaire (IPQ)^36^ (using 14 7-point Likert items ranging from strongly disagree to strongly agree) also once at the end of each condition. In line with previous work^37^, all questionnaires were filled in the VR environment to avoid breaks in presence. To prevent participants from moving their hands during the VR experiment, they completed the questionnaires using head movements. We used the position and rotation of the head-mounted display (HMD) and cast a ray in the game engine to determine participants’ responses. To familiarize participants with the questionnaires, we provided a tutorial prior to the experiment, during which they completed the questionnaires while seated on the bike.

### Apparatus

**Fig. 1:**
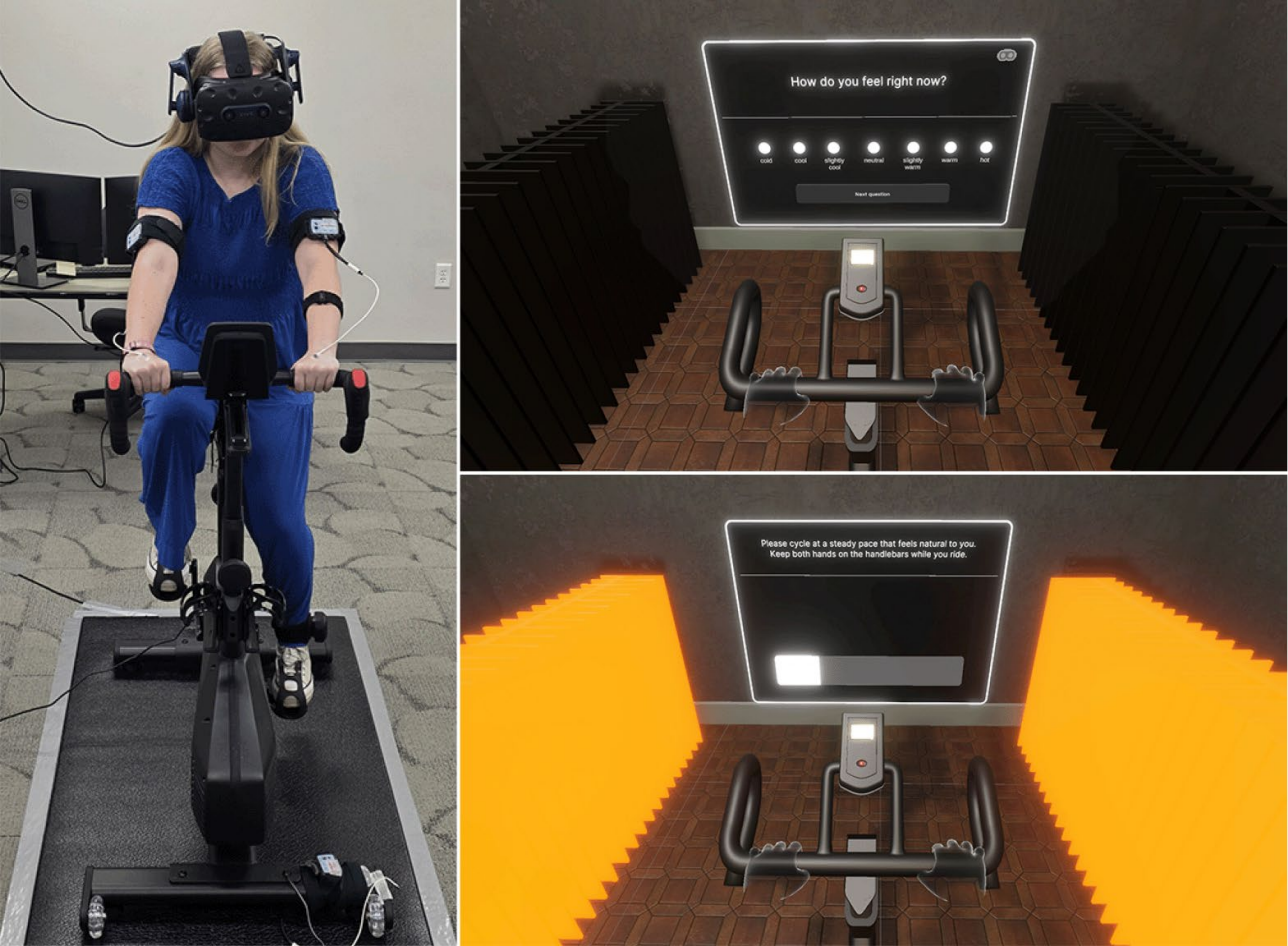
Left: Participant cycling on the stationary bicycle while wearing a VR device (HTC Vive Pro 2), skin temperature sensors attached to the back of both hands (BIOPAC skin temperature thermistors) connected with transmitters (BioNomadix SKT Transmitter) wrapped around the left and right upper arm. In addition, participants were wearing a heart rate sensor (Polar OH1) on the left forearm and an optical tracker (HTC Vive Ultimate Tracker) that was attached to their left foot to register and transfer the pedal motion onto the virtual replica of the ergometer in VR. Right: Deactivated (top) and activated (bottom) heater conditions during cycling in VR. Questionnaires were presented directly in front of participants (top) and could be completed using head movements. The screen on the virtual wall displayed the experimental progress using a slider that advanced continuously at a rate of one increment per second (bottom).

**Fig. 2:**
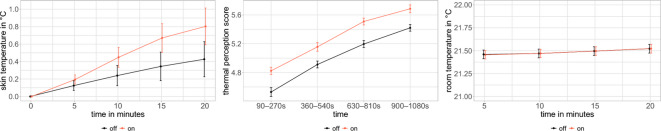
Normalized skin temperature averaged over five-minute intervals (left), absolute thermal sensation scores aggregated by consecutive triplets (center), and room temperature (right) for both virtual heater conditions (*off* and *on*). The error bars show the standard error.

We placed an electromagnetically braked bicycle ergometer (Wattbike Proton, Wattbike LTD, UK) in our VR laboratory. We used an air-conditioned VR laboratory to control the room temperature and ensure that both conditions are comparable (see Fig. 2). The laboratory did not contain visible heating, cooling, or ventilation devices, nor any other prominent objects commonly associated with warm or cool conditions. The ergometer was operated in watt mode, whereby resistance was automatically adjusted to maintain the target power output regardless of pedaling cadence, and, therefore, ensuring a cadence-independent workload. We used Unity (version 2022.3.14f1) to implement and design the VR application. We used and modified a 3D-model of the ergometer using the 3D-modelling software Blender. We also designed a virtual scene consisting of a fitness room with dark walls, the stationary bicycle, questionnaires and the virtual heaters (see Fig. 1). We used a HTC Vive Pro 2 VR Headset with a 2448 x 2448 pixels resolution per eye and a 120$^\circ$ field of view. The HMD was connected via cable to a workstation (MSI Codex Z2, Windows 11, AMD R7-8700F, Nvidia GeForce RTX 5070, 32GB DDR5 Ram), which ran the VR application and the software (GoldenCheetah, v 3.7.1) that connected the workstation with the bike (via ANT+) to control the Watt and record HR data. An HTC Vive Ultimate Tracker was attached to participants’ left foot to register and transfer the pedal motion onto the virtual replica of the ergometer in the virtual environment. Hence, participants experienced cycling the bike in VR.

Skin temperature was measured using two fast-response thermistors (TSD202B Skin Temperature Thermistor, BIOPAC, USA) attached to the backs of the participants’ hands, one on each hand. The experimenter cleaned the back of each hand with sterile skin-prepping wipes and used hypoallergenic adhesive tape to attach the senors onto the hands. In addition, a third fast-response thermistor was mounted on the foot of the bike to measure room temperature. The sensor was positioned in the air and did not come into contact with any surface. The thermistors were connected to the BioNomadix SKT Transmitter (BN-SKT2, BIOPAC, USA) to wirelessly transmit the data to the Smart Center module (BIOPAC, USA) that was connected to a laptop (Acer Nitro V16S, Windows 11, Intel 10-core Core 7 240H, 32GB RAM, Nvidia GeForce RTX 5060). Eventually, the laptop ran the AcqKnowledge Data Acquisition and Analysis Software (BIOPAC, USA) to continuously record the skin and room temperature data at 2000 Hz. Given the slow temporal dynamics of skin temperature, which typically varies on the order of seconds to minutes in thermophysiological responses^38,39^, the data were resampled to 1 Hz for analysis in line with previous work^13,18,19^. We measured participants’ HR using an optical HR monitor worn on their left arm (Polar OH1, Polar Electro, Finland). Previous work validated the Polar OH1 HR sensor as a valid measurement device during moderate and high intensity physical activities^40^. We continuously measured the HR throughout the experiment. The study was conducted indoors using air conditioning to maintain a stable room temperature for all participants and, therefore, ensuring comparable conditions.

**Table 1:** Distribution of cycling intensity in [W] based on participants’ body weight in kg, and the number of participants cycling with the respective intensities (N).

Weight (kg)	Intensity [W]	N
50–60	15	15
65–75	20	10
80–95	25	8
*>100*	30	1

The intensities were selected based on the standardized protocol by Northridge et al.^27^.

### Procedure

Upon arrival, participants’ verbal consent was obtained using an informed consent form. Eligibility was confirmed using the PAR-Q+ questionnaire^41^ to determine if participants were ready for physical activity. Before starting the experiment, the participants could change their clothes and put on sportswear in a separate room in our laboratory. Participants’ body weight and height were measured using a digital scale and height rod to determine exercise intensity relative to body weight. Age was also recorded to estimate maximum heart rate using the age-predicted $HR_{max}$ equation (i.e., $208 - 0.7 \times \text {age}$)^24^. Participants then completed a pre-cycling questionnaire assessing demographic information, prior experience with virtual reality and games, perceived suitability of their clothing for physical activity, and self-reported fitness.

Next, participants received an introduction to the VR equipment and study task. They were seated on the stationary ergometer and adjusted the seat height so that their knees remained slightly bent at the lowest pedal position. The experimenter then attached the heart rate monitor on the forearm and the skin temperature sensors secured to the backs of both hands. A VR motion tracker was attached to the participant’s left ankle to capture pedaling motion. Participants were fitted with the HMD and completed a tutorial to familiarize themselves with the VR environment and the method for answering questionnaires within VR, such as the RPE scale. In line with the Steep Bicycle Protocol proposed by Northridge et al.^27^, exercise intensity was normalized by body weight (e.g., 15 W for 50 kg participants and 30 W for 100 kg participants; see Table 1 for the distribution of Watts per body weight for all participants) and controlled via a software (GoldenCheetah) running on the workstation. Accordingly, participants cycled on the stationary ergometer for 20 minutes in each condition.

During cycling, participants reported their perceived exertion using the RPE Scale^35^ and rated their thermal perception using questionnaires presented in VR. After each cycling session, participants completed additional questionnaires within VR assessing thermal perception and sense of presence in the virtual environment while remaining seated on the bike without pedaling. This phase was used as a three-minute resting period. If they completed the questionnaires before 3 minutes had passed, a loading bar was spawned, indicating when the next condition starts. This allowed controlling the duration of each condition to recalibrate participants’ thermal perception and body temperature. The procedure was then repeated for the second condition. At the end of the experiment, physiological recording was stopped and all sensors and VR equipment were removed. Participants were then debriefed and the equipment sanitized. The experiment took approximately 75 minutes per participant in total, of which around 46 minutes were spent immersed in VR.

### Statistical analysis

To account for time-related trends and repeated measures, separate linear mixed-effects models (LMMs) were fitted for skin temperature and thermal sensation as well as heart rate, perceived exertion, and cadence. Random-effects structures were determined individually for each outcome based on model convergence. Heater (*off* vs. *on*) was treated as fixed factor in all analyses. For continuous measures (skin temperature, heart rate, and cadence), Time was modeled as a numeric predictor (after *5, 10, 15, and 20* min). Minute 0 served as an individualized baseline and was excluded from the model. For discrete repeated measures (thermal perception and perceived exertion), Time was specified as an ordinal factor (*90-270s*, *360-540s*, *630-810s*, and *900-1080s*). We used R to analyze the data (version 4.5.1, R Core Team^42^). We averaged the measures at five-minute intervals to obtain the time course across the entire conditions.

## Results

In the following, we report the results for each measure individually, outlining the observed effects and trends for both perceptual responses and physiological indicators.

### Skin temperature

We averaged temperature data at five-minute intervals. Minute 0 served as an individualized baseline and was excluded from the model. Random-effects structures were initially specified to include random slopes for Heater and Time. However, models including random slopes for Time resulted in singular fits, indicating that the data did not support estimation of between-subject variability in time trends. Therefore, the final models included random intercepts and random slopes for Heater only. Figure 2 shows mean normalized skin temperature responses as a function of Heater *×* Time. The LMM revealed no significant main effect of Heater, *b = -0.014*, *SE = 0.247*, *t(59.73) = -0.06*, *p =.955*. There was a significant main effect of Time, *b = 0.020*, *SE = 0.007*, *t(202) = 2.82*, *p =.005*, indicating that the skin temperature increased over time. Importantly, the Heater *×* Time interaction was also significant, *b = 0.021*, *SE = 0.010*, *t(202.00) = 2.04*, *p =.042*, suggesting that the change in skin temperature over time differed depending on whether the heater was on or off. This indicates that skin temperature significantly increased more rapidly in the Heater *on* condition compared to the Heater *off* condition. Descriptive statistics show that after 5 minutes of cycling, skin temperature increased on average by $0.19^\circ \textrm{C}$ (*SD = 0.31*) in the activated heater condition, compared to $0.12^\circ \textrm{C}$ (*SD = 0.32*) in the deactivated condition (*Δ t* (on–off) $= 0.07^\circ \textrm{C}$). After 20 minutes of cycling, skin temperature increased on average by $0.80^\circ \textrm{C}$ (*SD = 1.23*) in the activated heater condition, compared to $0.43^\circ \textrm{C}$ (*SD = 1.17*) in the deactivated condition (*Δ t* (on–off)$= 0.37^\circ \textrm{C}$). This indicates a steeper slope for the Heater *on* condition compared to the the Heater *off* condition.

### Thermal sensation

Thermal sensation scores were aggregated into consecutive triplets of measurements: 90–270 s (90, 180, 270 s), 360–540 s (360, 450, 540 s), 630–810 s (630, 720, 810 s), and 900–1080 s (900, 990, 1080 s). Time was added as a factor to the analysis. Figure 2 shows average thermal sensation scores as a function of Heater *×* Time. The LMM revealed a significant main effect of Heater, *b = 0.29*, *SE = 0.15*, *t(231) = 2.01*, *p =.045*, indicating higher values in the heater-on condition compared to the heater-off condition. There was also a significant main effect of Time, with increases relative to the reference time point at 360–540 s (*b = 0.38*, *SE = 0.15*, *t(231) = 2.62*, *p =.010*), 630–810 s (*b = 0.67*, *SE = 0.15*, *t(231) = 4.56*, *p <.001*), and 900–1080 s (*b = 0.89*, *SE = 0.15*, *t(231) = 6.10*, *p <.001*). The Heater *×* Time interaction was not significant (all *|t| łe 0.24*, all *p \ge .813*), indicating that the effect of the heater did not vary across time. Descriptive statistics show that participants felt slightly but consistently warmer in the activated heater condition (during 900-1080s, *M = 5.69*, *SD = 0.31*) compared to the deactivated condition (during 900-1080s, *M = 5.42*, *SD = 0.27*).

### Thermal comfort

Thermal comfort was assessed once at the end of each condition. A paired-samples *t*-test revealed no significant effect of Heater on thermal comfort, *t(33) = 0.45*, *p =.656*. While descriptive statistics show a lower thermal comfort in terms of increased heat for the Heater *on* condition (*M = 0.73*, *SD = 1.29*) compared to the Heater *off* condition (*M = 0.59*, *SD = 1.21*), their thermal comfort did not differ significantly between conditions.

### Perceived exertion

**Fig. 3:**
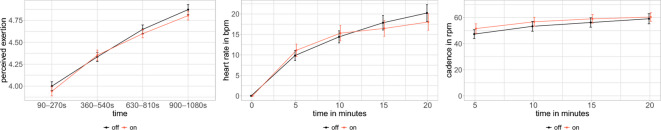
Perceived exertion scores aggregated by consecutive triplets (left), normalized heart rate (center), and pedaling cadence (right) for both virtual heater conditions (*Off* vs. *On*). The error bars show the standard error.

Perceived exertion scores were aggregated into consecutive triplets of measurements: 90–270 s (90, 180, 270 s), 360–540 s (360, 450, 540 s), 630–810 s (630, 720, 810 s), and 900–1080 s (900, 990, 1080 s). Time was added as a factor to the analysis. Figure 3 shows average perceived exertion scores as a function of Heater *×* Time. The LMM revealed no significant main effect of Heater, *b = -0.06*, *SE = 0.15*, *t(231) = -0.39*, *p =.699*, indicating no difference between the heater-on and heater-off conditions. There was, however, a significant main effect of Time, with increases relative to the reference time point at 360–540 s (*b = 0.33*, *SE = 0.15*, *t(231) = 2.19*, *p =.029*), 630–810 s (*b = 0.65*, *SE = 0.15*, *t(231) = 4.25*, *p <.001*), and 900–1080 s (*b = 0.87*, *SE = 0.15*, *t(231) = 5.74*, *p <.001*). The Heater *×* Time interaction was not significant (all *|t| łe 0.45*, all *p \ge .715*), indicating that the effect of the heater did not vary across time.

### Cadence

We averaged pedaling cadence data at five-minute intervals. Minute 0 served as an individualized baseline and was excluded from the model. Random intercepts and slopes for Heater were specified for each participant. Figure 3 shows the mean pedaling cadence as a function of Heater *×* Time. The LMM revealed no significant main effect of Heater, *b = 5.343*, *SE = 2.674*, *t(59.48) = 1.99*, *p =.050*. There was a significant main effect of Time, *b = 0.764*, *SE = 0.078*, *t(202.00) = 9.85*, *p <.001*, indicating that pedaling cadence increased over time. The Heater *×* Time interaction was also not significant, *b = -0.189*, *SE = 0.110*, *t(202.00) = -1.72*, *p =.086*, suggesting that the change in pedaling cadence over time did not differ between heater conditions.

### Heart rate

We averaged heart rate data at five-minute intervals. Minute 0 served as an individualized baseline and was excluded from the model. No random intercepts and slopes were specified. Figure 3 shows mean normalized heart rate responses as a function of Heater *×* Time. The LMM revealed no significant main effect of Heater, *b = 2.821*, *SE = 2.033*, *t(235) = 1.39*, *p =.166*. There was a significant main effect of Time, *b = 0.691*, *SE = 0.10*, *t(235) = 6.586*, *p <.001*, indicating that heart rate increased over time. The Heater *×* Time interaction was not significant, *b = -0.254*, *SE = 0.148*, *t(235) = -1.71*, *p =.089*.

### Presence

**Fig. 4:**
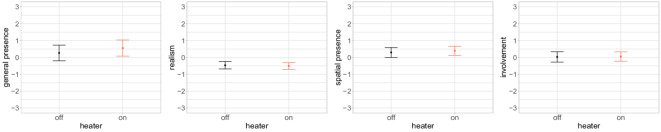
IPQ’s^36^ general presence, realism, spatial presence, and involvement dimensions. The error bars show the standard error.

We analyzed each dimension of the IPQ^36^, namely general presence, realism, spatial presence, and involvement. Figure 4 depicts the mean IPQ scores for each dimension as a function of Heater. Independent *t*-tests revealed no significant effects of Heater on any of the IPQ dimensions (all *p >.05*). These findings indicate that participants’ sense of presence did not significantly differ between heater conditions.

## Discussion

This study investigated whether visual thermal cues influence thermophysiological and perceptual responses during a physical exercise in VR. Our results show that activating a virtual heater increased both skin temperature and thermal perception. The findings extend prior work on the hue-heat hypothesis to dynamic exercise contexts and suggest that visual cues can modulate thermoregulation even when strong interoceptive signals are present.

The observed skin temperature increase in the heater condition indicates that visual thermal cues can affect peripheral thermophysiological responses during exercise. As the room temperature was controlled in an air-conditioned laboratory (see Fig. 2), we can rule out actual changes in ambient temperature as the cause of the observed effects. One possible explanation is that the visual representation of the heater elicited associations with warmth, as reflected in elevated perceived temperature. These induced thermal associations influenced expectations about environmental temperature^12,18^. This, in turn, triggered thermoregulatory mechanisms such as vasodilation, i.e., increased peripheral blood flow to the hands to cool the body by facilitating heat dissipation^12,13,43,44^. This explanation is supported by the hue–heat hypothesis^10,45^ and advances the current state of knowledge by demonstrating that the hue-heat hypothesis also occurs during physical activity.

Notably, the effect emerged over time, as reflected in the significant Heater *×* Time interaction and increased throughout the 20 minutes of exposure (see Fig. 2). This suggests that visual cues do not produce an immediate physiological response but gradually modulate thermoregulation during sustained activity. While not directly comparable to prior work demonstrating changes in skin temperature induced by visual cues during sedentary experiences^12,13,18^, our findings align with evidence suggesting that such thermophysiological effects increase over time. Wührl et al.^19^ reported changes in skin temperature after five and ten minutes of exposure to fire and ice virtual environments during a seated VR experience. Extending this line of work, our results indicate that these effects persist for at least 20 minutes. Moreover, the observation of similar time-dependent effects during dynamic physical activity, such as cycling, highlights the robustness and persistence of thermophysiological responses elicited by visual thermal cues.

The hue–heat hypothesis proposes that warm visual cues can influence thermal perception, thereby eliciting autonomic physiological responses. This suggests that thermoregulatory processes are not driven solely by sensory feedback from temperature receptors in the body (e.g., the skin), but also involve anticipatory feedforward mechanisms. Although a recent review by Mitchell et al.^46^ challenges this notion, the authors nevertheless conclude that both feedforward mechanisms and feedback mechanisms, i.e., where receptors detect physiological changes and relay signals to thermoregulatory control centers, contribute to thermoregulation. However, they argue that feedback control represents the dominant regulatory process. In our study, only an activated or deactivated virtual heater was presented while the physical ambient temperature remained unchanged. Consequently, thermoreceptors could not initially detect any temperature differences, implying that any early thermoregulatory responses must have been triggered by feedforward mechanisms. Over time, however, these responses may have induced physiological adaptations, such as vasodilation and increased blood flow to the hands, resulting in actual changes in skin temperature. Once such physiological changes occurred, they could have been detected by thermoreceptors, thereby engaging feedback control processes. Accordingly, our findings suggest that feedforward mechanisms may initiate thermoregulatory responses, which are subsequently reinforced or modulated through feedback mechanisms. Future research could leverage VR to systematically investigate how, when, and to what extent feedforward and feedback processes interact during thermoregulation.

Another possible explanation for the observed effects on skin temperature is behavioral adaptation induced by the virtual heater. Although exercise performance was controlled using watt mode, i.e., resistance dynamically adjusts to maintain a constant power output independent of cadence, variations in cadence may still influence skin temperature^47^. However, our analysis revealed no significant differences between heater conditions, and the descriptive statistics support this observation (see Fig. 3). While we rule out that changes in skin temperature were driven by cadence differences, future work could further explore behavioral changes induced by virtual thermal cues, e.g., different body movement while cycling in response to increased perceived temperature.

In addition, we included a three-minute resting period between conditions to allow participants’ thermal sensations and physiological responses to return closer to baseline. Because the study employed a within-participants design involving prolonged physical activity, baseline skin temperatures were slightly higher on average in the second condition than in the first. Participants showed baseline temperatures of *M* = 29.95$^\circ$C, *SD* = 1.96$^\circ$C when receiving the activated heater as the first condition and *M* = 31.08$^\circ$C, *SD* = 1.77$^\circ$C as the second condition. Similarly, participants showed baseline temperatures of *M* = 30.10$^\circ$C, *SD* = 1.66$^\circ$C when receiving the deactivated heater as the first condition and *M* = 31.43$^\circ$C, *SD* = 1.92$^\circ$C as the second condition. However, because condition order was fully counterbalanced and all condition sequences were administered equally often, we rule out that the observed Heater *×* Time interaction on skin temperature can be attributed to order effects.

An additional factor to consider in relation to thermoregulation is participants’ clothing. Participants wore their own sportswear during the cycling exercise and could change clothes in a separate room before the experiment. We did not provide standardized sportswear to maintain ecological validity and better reflect real-world exercise conditions. Although differences in clothing may have introduced additional variability in the data, using participants’ own sportswear increases the generalizability of the findings. Importantly, despite variation in clothing characteristics, we observed a consistent effect on skin temperature over time. This suggests that the effect of the virtual heater was robust enough to be detected under realistic conditions.

In contrast to the effects on skin temperature, the effects on thermal sensation did not significantly increase over time. This is also in line with Wührl et al.^19^ who did not find an interaction effect of thermal cues and time on thermal sensation while being immersed in a fire and ice world. As expected, our results suggest that thermal sensation increases over time while cycling. However, this increase is independent of the heater condition (see Fig. 2). Thus, we assume that subjective effects manifest from the very beginning of the exposure. Effects on skin temperature, however, slowly increase as thermoregulation in the human body slowly adapts^1,6^. Confirming H1, participants reported feeling warmer in the activated heater condition than in the deactivated heater condition. Thermal sensation was assessed using a standardized scale^33^, which captures overall thermal sensation rather than localized perceptions of warmth or heat. From an applied perspective, whole-body thermal sensation is particularly relevant during cycling, as it reflects participants’ overall thermal experience. Nevertheless, assessing localized thermal sensations, for example at the hands, could provide additional insights into the subjective effects of localized heating or cooling and help clarify how these perceptions relate to physiological responses. Future research could further extend these findings by employing infrared thermography to capture spatial patterns of skin temperature across the body, thereby enabling a more comprehensive assessment of localized and whole-body thermoregulatory responses.

Differently from what was expected, we did not observe statistically significant effects of the heater condition on perceived exertion and heart rate. This suggests that participants did not perceive the cycling exercise as more exhausting during the activated heater compared to the deactivated heater. Descriptive statistics confirm this trend (see Fig. 3). Hence, we reject H2. One possible explanation is that an increase in perceived warmth does not automatically translate into an increase in perceived exertion or vice versa. Roussey at al.^2^, for example, showed that participants experienced an increased perceived warmth despite a steady perception of effort. This is in line with Heydenreich et al.^48^ who summarized in a meta analysis that perceived exertion reflects an integration of multiple psychophysiological signals, e.g., heart rate, and does not increase in a fixed one-to-one manner with perceived warmth^49,50^. This pattern is also reflected in the thermal comfort ratings. Although participants reported higher thermal sensation (i.e., feeling warmer), thermal comfort did not differ significantly between conditions. This indicates that the increased perceived warmth did not translate into reduced comfort. Accordingly, neither perceived exertion nor thermal comfort, both subjective measures of comfort, were influenced by the activation state of the virtual heater. To enhance these effects, future work could explore alternative designs of thermal cues. For instance, a virtual heater could incorporate additional visual elements such as particle effects (e.g., heat shimmer), as well as auditory cues that reinforce common associations with warmth, e.g., a subtle electrical sound upon activation or, in the case of a fire-based heater, the crackling of flames.

A similar dissociation of subjective and objective measure was found for skin temperature and perceived exertion. Levels et al.^51^ found that athletes cycling a 7.5-km time trial with higher mean skin temperatures did not report greater perceived exertion compared to those with lower skin temperatures. The authors suggest that the duration of exercise may be critical, as the effects of elevated skin temperature may exhibit a delayed onset. In their study, participants cycled for approximately 12 minutes. In contrast, Tucker et al.^52^ demonstrated that skin temperature influences both the selection of exercise intensity and perceived exertion during longer trials lasting between 34 and 50 minutes. In our study, participants cycled for 20 minutes per condition. Together, these findings suggest that the effects of elevated skin temperature require sufficient time to develop and manifest in increased perceived exertion and the corresponding cardiovascular response. Hence, future work should explore longer exercises to learn more about the time-dependence and robustness of the effects during dynamic physical activity.

Our findings highlight that differences in skin temperature are not driven by physical or psychological workload. Participants exhibited increased skin temperature despite no corresponding changes in perceived exertion or heart rate. This challenges a potential causal interpretation in which a virtual heater first increases perceived warmth, leading to elevated perceived exertion and heart rate, which in turn raise skin temperature. Instead, our results indicate that neither perceived exertion nor cardiovascular responses account for the observed increase in skin temperature over time. Rather, the effects appear to be directly induced by the visual thermal cue itself, i.e., the activated virtual heater. This further underscores the robustness of the hue–heat hypothesis, even in dynamic contexts involving physical activity.

Future work should also examine these effects across different exercise intensities. In the present study, we employed a low wattage protocol, resulting in low to moderate perceived exertion (on a scale from 1 = very light to 10 = maximal effort; aggregated across heater conditions and time: *M = 4.44*, *SD = 1.15*), to ensure participants remained within the aerobic threshold (approximately 70% of maximum heart rate) and below the lactate turn point^29^. This approach enhanced comparability between conditions and minimized confounding effects from behavioral adaptations associated with higher intensities (e.g., standing on the pedals or increased body movement). However, this study does not address how these effects manifest during high-intensity exercise. We hypothesize that exercise intensity modulates the magnitude of the effect. Specifically, at cycling intensities exceeding the anaerobic threshold, the influence of visual thermal cues may be attenuated, as dominant interoceptive signals shift attention away from the external environment and toward internal bodily responses. Future research should therefore investigate a broader range of exercise intensities to better understand the sustainability and applicability of these effects during more demanding physical activity. Besides, a systematic comparison of the effects of visual thermal cues on perceptual and physiological responses during rest and higher levels of physical activity could also provide valuable insights into the magnitude of these effects and their relative contribution under different levels of physical strain. Hence, future research could directly compare the effects predicted by the hue–heat hypothesis during resting and cycling conditions.

Another promising avenue for future research is to further investigate the mechanisms through which visual thermal cues influence thermal sensation. For example, Filingeri et al.^53^ examined how perceived skin wetness and thermal perception interact during rest and cycling under different ambient temperatures (22$^\circ$C and 33$^\circ$C). Their findings suggest that thermal sensations can influence perceived skin wetness, with colder sensations leading participants to perceive their skin as wetter. Such mechanisms may also be relevant to the effects observed in the present study. Although sweating responses were not assessed, future work could examine whether exposure to a deactivated virtual heater induces colder thermal sensations, which in turn increase perceived skin wetness during exercise and thereby enhance the sensation of sweating. Related research has systematically manipulated sweating-related cues through virtual avatars that appeared either sweaty or non-sweaty^54,55^. These studies indicate that sweaty avatars can reduce perceived exertion and increase perceived endurance. However, it remains unclear whether visual thermal cues can similarly modulate perceived or actual sweating responses. Investigating the interplay between visual thermal cues, perceived wetness, sweating, and thermal sensation could therefore provide valuable insights into the mechanisms underlying these effects.

Measuring core body temperature could help provide a more comprehensive picture of the thermophysiological responses. While we followed prior work^18,31,32^ by measuring hand temperature as a sensitive indicator of thermoregulatory responses, core body temperature could offer a more direct measure of changes in thermoregulation. Increases in hand temperature are thought to reflect peripheral vasodilation, a mechanism through which the body dissipates heat by increasing blood flow to the extremities^12,13,18,19^. Given that we only used visual thermal cues and did not alter the actual ambient temperature, examining whether such visually induced responses are accompanied by measurable changes in core temperature could provide further insights into thermoregulation. Future studies could therefore extend our findings by incorporating non-invasive core temperature measurements, for example using heat-flux-based sensors that accurately estimate core body temperature^56^.

## Conclusion

In this paper, we investigate the effects of visual thermal cues on perceptual and physiological responses during physical exercise. In a controlled VR experiment, 34 participants cycled for 20 minutes while a virtual heater was either activated or deactivated. Our results show that an activated virtual heater led to a greater increase in skin temperature over time compared to the deactivated condition. In addition, participants’ thermal sensation was significantly influenced by the heater’s activation state, with participants reporting feeling slightly warmer when the virtual heater was active. Overall, our findings demonstrate that visual thermal cues can influence not only subjective thermal perception but also thermoregulatory responses, as evidenced by changes in skin temperature. This work extends prior research by showing that such effects even persist during lower-intensity physical activity. Future research may build on these findings by examining longer-term effects and investigating how these responses evolve under higher exercise intensities. Such work could further advance our understanding of human thermoregulation and temperature perception in visually augmented exercise environments.

## Data Availability

The datasets generated during and/or analyzed during the current study are available from the corresponding author on request.
